# Oxidative stress and mitochondrial dynamics malfunction are linked in Pelizaeus‐Merzbacher disease

**DOI:** 10.1111/bpa.12571

**Published:** 2017-12-26

**Authors:** Montserrat Ruiz, Mélina Bégou, Nathalie Launay, Pablo Ranea‐Robles, Patrizia Bianchi, Jone López‐Erauskin, Laia Morató, Cristina Guilera, Bérengère Petit, Catherine Vaurs‐Barriere, Céline Guéret‐Gonthier, Marie‐Noëlle Bonnet‐Dupeyron, Stéphane Fourcade, Johan Auwerx, Odile Boespflug‐Tanguy, Aurora Pujol

**Affiliations:** ^1^ Neurometabolic Diseases Laboratory Bellvitge Biomedical Research Institute (IDIBELL), 08908 L'Hospitalet de Llobregat Barcelona Spain; ^2^ Center for Biomedical Research on Rare Diseases (CIBERER), ISCIII Spain; ^3^ Inserm, UMR 1107, NEURO‐DOL F‐63001 Clermont‐Ferrand France; ^4^ Université Clermont Auvergne, NEURO‐DOL, BP 10448 F‐63000 Clermont‐Ferrand France; ^5^ Université Clermont Auvergne, GReD, BP 10448 F‐63000 Clermont‐Ferrand France; ^6^ CHU de Clermont Ferrand, Laboratoire de cytogénétique F‐63000 Clermont‐Ferrand France; ^7^ Laboratory for Integrative and Systems Physiology École Polytechnique Fédérale de Lausanne, Station 15 CH‐1015 Lausanne Switzerland; ^8^ Assistance Publique des Hopitaux de Paris (APHP), Reference Center for Rare Diseases “Leukodystrophies,” Child Neurology and Metabolic Disorders Department Robert Debré University Hospital Paris France; ^9^ Inserm, Paris Diderot University UMR 1141, DHU PROTECT, Sorbonne Paris‐Cite Robert Debré University Hospital Paris France; ^10^ Institute of Neuropathology University of Barcelona, L'Hospitalet de Llobregat Barcelona Spain; ^11^ Catalan Institution of Research and Advanced Studies (ICREA) Barcelona Spain

**Keywords:** antioxidants, bioenergetic failure, mitochondrial dynamics, oxidative stress, Pelizaeus‐Merzbacher disease

## Abstract

Pelizaeus‐Merzbacher disease (PMD) is a fatal hypomyelinating disorder characterized by early impairment of motor development, nystagmus, choreoathetotic movements, ataxia and progressive spasticity. PMD is caused by variations in the proteolipid protein gene *PLP1*, which encodes the two major myelin proteins of the central nervous system, PLP and its spliced isoform DM20, in oligodendrocytes. Large duplications including the entire *PLP1* gene are the most frequent causative mutation leading to the classical form of PMD. The Plp1 overexpressing mouse model (PLP‐tg^66/66^) develops a phenotype very similar to human PMD, with early and severe motor dysfunction and a dramatic decrease in lifespan. The sequence of cellular events that cause neurodegeneration and ultimately death is poorly understood. In this work, we analyzed patient‐derived fibroblasts and spinal cords of the PLP‐tg^66/66^ mouse model, and identified redox imbalance, with altered antioxidant defense and oxidative damage to several enzymes involved in ATP production, such as glycolytic enzymes, creatine kinase and mitochondrial proteins from the Krebs cycle and oxidative phosphorylation. We also evidenced malfunction of the mitochondria compartment with increased ROS production and depolarization in PMD patient's fibroblasts, which was prevented by the antioxidant N‐acetyl‐cysteine. Finally, we uncovered an impairment of mitochondrial dynamics in patient's fibroblasts which may help explain the ultrastructural abnormalities of mitochondria morphology detected in spinal cords from PLP‐tg^66/66^ mice. Altogether, these results underscore the link between redox and metabolic homeostasis in myelin diseases, provide insight into the pathophysiology of PMD, and may bear implications for tailored pharmacological intervention.

## Introduction

Pelizaeus‐Merzbacher Disease (PMD) is the prototype disease for an inherited defect in CNS myelin formation. This X‐linked form of hypomyelinating leukodystrophy (HLD1, OMIM 312080) is caused by mutation of the proteolipid protein gene (*PLP1* in Xq22.2), which is expressed in oligodendrocytes. *PLP1* encodes the two major myelin proteins of the central nervous system (CNS), PLP and its spliced isoform DM20. PLP is a major structural component of CNS myelin whereas DM20, which is produced earlier in CNS development, may be involved in oligodendrocyte differentiation and survival. The clinical findings associated with *PLP1* mutations are spread over a continuous spectrum extending from the most severe form of PMD to the relatively mild late‐onset type 2 spastic paraplegia, which led to the concept of *PLP1*‐related disorders 10. There is a phenotype‐genotype correlation: in PMD, overexpressed or misfolded PLP is retained in the endoplasmic reticulum where it interferes with oligodendrocytes survival or myelin production, whereas loss of PLP results in type 2 spastic paraplegia, which undergoes with length‐dependent axonal degeneration secondary to the abnormal myelin compaction. Large duplications including the entire *PLP1* gene are the causative mutation in 60% of PMD 10.

PMD is characterized in male patients by an early impairment of psychomotor development (< 6 months of life). During the first years of active myelination, this hypotonia is associated with neurological signs that are gradually modified by the maturing nervous system (nystagmus, choreoathetotic movements and ataxia). The best motor function achieved between the ages of 2 and 4 years classifies the patient according to disease severity 10. PMD forms 1 and 2 with, respectively, achievement of head control and sitting position are the most frequent, particularly in patients with *PLP1* duplications. Magnetic resonance imaging is used to diagnose the defect in CNS myelin formation by both dramatic and extensive abnormalities of multimodal evoked potentials in the CNS, and the diffuse hypomyelinated pattern of the subtentorial white matter 71.

Progressive spastic paraplegia with pyramidal tract signs and decrease in growth rate is subsequently observed leading to severe quadriplegia, amyotrophia and optic atrophia at the end of the second decade and accompanied by the development of cortico‐subcortical atrophy on magnetic resonance imaging 8. No treatment is available except for symptomatic medication against abnormal movements and spasticity. Survival up to the third or even fourth decade with severe handicap is commonly reported.

The sequence of cellular events that cause the severe neurological dysfunction and ultimately death in PMD patients is poorly understood. PLP‐tg^66/66^ is a relevant model to investigate the pathophysiology of classical PMD (forms 1 and 2) 61. These mice exhibit early and severe motor dysfunctions (from the second week of age), similar to humans. Subsequently, a dramatic growth failure associated with seizures/dystonic crisis is observed around 1 month of age, leading to death before the age of 3 months. A marked hypomyelination is associated with an increased number of apoptotic oligodendrocytes 33, 61. Myelin sheath thickness is largely decreased in all CNS structures and totally disappears as the disease progresses.

In different animal models of demyelination, increased numbers of intra‐axonal mitochondria have been described 2, 28, 51, 63, most commonly attributed to an axonal adaptive process to preserve conduction after demyelination 80. Conversely, primary mitochondrial abnormalities have been shown to cause severe myelin damage 47, 64. In the brain of PLP‐tg^66/66^ mice, a decrease in ATP levels and mitochondrial membrane potential associated with an increase both in cytochrome c oxidase staining and in the number and size of mitochondria in oligodendrocytes was reported. These defects were associated with abnormal co‐localization of PLP to the mitochondrial membrane 3, 29. However, the potential impact of this increased mitochondrial density and activity on redox homeostasis, and thus, a role for oxidative stress in disease pathogenesis, is unknown.

In the present study, we demonstrate that PLP‐tg^66/66^ mice spinal cords have increased size of mitochondria in both axons and oligodendrocytes cytoplasm associated with increased free radical production and oxidative damage to proteins involved in bioenergetic metabolism. This is concomitant with decreased levels of ATP and altered enzymatic antioxidant defenses. Interestingly, the increase in both the number and density of mitochondria, and levels of proteins involved in mitochondrial oxidative phosphorylation, as well as an increase in pyruvate kinase (PK) activity are concomitant with an alteration of the mitochondrial fission process, suggesting a main role of mitochondria dynamics and subsequent mitochondria dysfunction in the pathogenesis of PMD. These findings provide new therapeutic targets and open the door to mitochondria‐targeted antioxidant treatments already tested in neurological disorders accompanied by axonal degeneration 22, 41, 59, 70, 87.

## Materials and Methods

### Mouse breeding

*Plp1* overexpressing mice from line #66 and their wild‐type littermates were generated as previously described 61, and carry 14 supplementary copies of a *Plp1* genomic transgene in homozygosis. PLP‐tg^66/66^ mice were bred into the C57BL/6N background using mice from the breeding colony of the Max Planck Institute of Experimental Medicine and then transferred to the University of Auvergne animal facility (kind gift from Pr K.A. Nave, Göttingen, Germany). Genotyping was performed as previously described 61. Mice were housed eight per cage in a temperature (22°C) controlled environment under a 12:12 light/dark cycle (light from 7:00 AM to 7:00 PM), with *ad libitum* access to food and water.

The study was performed in compliance with European legislative, administrative and statutory provisions for the protection of animals used for experimental or other scientific purposes (2010/63/UE).

### Chemicals

The following chemicals were used: DCF (Molecular probes, Life Technologies, Waltham, Massachusetts), DHE (Molecular Probes), MitoSOX™ (Molecular Probes), NAC (Sigma, Sigma‐Aldrich, St. Louis, Missouri), TMRE (Invitrogen, Life Technologies, Waltham, Massachusetts) and CCCP (Sigma). All other reagents were from Sigma‐Aldrich Spain, of the highest purity available.

### Antibodies

The following antibodies were used for western blots: anti‐rabbit DNP (dilution 1:500; D9659, Sigma); anti‐rabbit Glutamine Synthetase (dilution 1:2000; G2781, Sigma); anti‐rabbit Phosphoglycerate kinase 1 (dilution 1:500; ab38007, Abcam, Cambridge, UK); anti‐mouse Enolase 1 (dilution 1:2000; H00002023‐M01, Abnova, Taipei City, Taiwan); anti‐mouse GAPDH (dilution 1:10 000; AM4300, Ambion, Thermo Fisher Scientific, Inc., Waltham, Massachusetts); anti‐goat Aldolase A (dilution 1:2000; NB600–915, Novus Biologicals, Saint Charles, Missouri); anti‐rabbit GPX1 (dilution 1:500; LF‐PA0087, Abfrontier Seoul, South Korea); anti‐rabbit Catalase (dilution 1:2000; 200–4151, Rockland, Limerick, Pennsylvania); SOD‐1 (dilution 1:500; NCL‐SOD1, Novocastra, Leica, Wetzlar, Germany); SOD2 (dilution 1:1000; 61 1581, BD Biosciences Pharmingen, Franklin Lakes, New Jersey); anti‐rabbit Glutathione reductase (dilution 1:2000; ab16801, Abcam); anti‐rabbit PK M2 (dilution 1:500; ab38237, Abcam); anti‐goat Aspartate aminotransferase 2 (dilution 1:5000; AA295–306, Sigma); anti‐mouse Complex I‐NDUFA9 (dilution 1:1000; A21344, Invitrogen), anti‐mouse Complex I‐NDUFB8 (dilution 1:2000; 459 210, Invitrogen); anti‐mouse Complex II‐subunit 30 kDa (dilution 1:400; 459 230, Invitrogen); anti‐mouse Complex III‐subunit Core I (dilution 1:2000; 459 140, Invitrogen); anti‐mouse Complex IV‐subunit IV (dilution 1:2000; A21348, Invitrogen); anti‐mouse Complex V‐ subunit α (dilution 1:1000; 459 240, Invitrogen); anti‐rabbit VDAC1/Porin (dilution 1:2000; ab15895, Abcam); anti‐rabbit Aconitase 2 (dilution 1:500; AP1936c, Abgent, San Diego, California); anti‐rabbit Lipoamide dehydrogenase (dilution 1:500; L2498‐05, US Biological Salem, Massachusetts); anti‐mouse DRP1 (dilution 1:1000; 611 112, BD Transduction Laboratories, Franklin Lakes, New Jersey); anti‐rabbit Fis1 (dilution 1:1000; JM‐3491R‐100, MBL International Corporation, Woburn, Massachusetts); anti‐mouse OPA1 (dilution 1:1000; 612 607, BD Transduction Laboratories); anti‐rabbit Mitofusin 2 (dilution 1:1000; M6444, Sigma), anti‐mouse Tom20 (dilution 1:200; ab56783, Abcam) and anti‐mouse gamma‐Tubulin (dilution 1:10 000; T6557 clone GTU‐88, Sigma), incubated overnight at 4°C as primary antibodies. Goat anti‐rabbit, goat anti‐mouse, rabbit anti‐goat immunoglobulin G linked to horseradish peroxidase (dilution 1:15 000; P0448, P0447 and P0449, Dako Santa Clara, California) and anti‐mouse Alexa‐labelled secondary antibody (dilution 1:1000; A11001, Invitrogen) were used as secondary antibodies and incubated 1 h at room temperature.

### Spinal cord neuropathological analysis

For neuropathological analysis, mice were sacrificed under pentobarbitone anesthesia by perfusion (peristaltic pump, rate of 20 mL/minute for 5 minutes) through the left ventricle of freshly prepared in 0.1M PBS 2% paraformaldehyde and 2% glutaraldehyde fixative solution. Spinal cords were removed and kept overnight at 4°C in the fixative used for animal perfusion and then stored at 4°C in 4% paraformaldehyde during less than 1 month.

For semithin sectioning, sections from 4 PLP‐tg^66/66^ and 4 WT mice were extensively washed with sodium cacodylate and then post‐fixed in 1% osmium tetroxide for 1 h. Afterward, sections were dehydrated in alcohols and acetone. After embedding in epoxy resin, 700 nm thin coronal sections were performed and stained with toluidine blue. Semithin sections were examined with an Olympus Bx51 TF microscope (Olympus, Southend‐on‐Sea, UK) and digital images were captured with a CCD digital camera (Roper Scientific GmbH, Ottobrunn, Germany). Seventy‐nanometer ultrathin sections stained with uranyl acetate and lead citrate were also examined in a transmission electron microscope (Hitachi H7650, Tokyo, Japan), 80 kV. To characterize the mitochondrial population in the ventral column white and gray matter intermediate zone, the electron microscopy images were analyzed using the counting and area analysis function of the FIJI software. For each mouse (n = 3 WT and n = 3 PLP‐tg^66/66^), data have been obtained from 100 axons and 10 oligodendrocytes in both gray and white matter.

### Mono‐ and two‐dimensional electrophoresis and Western blotting

Electrophoresis and Western blotting were performed as previously described 23. Briefly, for mono‐dimensional Western blot frozen spinal cords were homogenized in radioimmunoprecipitation assay buffer and sonicated for 2 minutes at 4°C. Proteins of 30–50 mg were separated in polyacrylamide gels and transferred onto nitrocellulose membranes. Proteins were detected with ECL Western blotting analysis system followed by exposure to CL‐XPosure Film (Thermo Scientific, Thermo Fisher Scientific, Inc., Waltham, Massachusetts). Autoradiographs were scanned and quantified using a GS800 Densitometer and Quantity One software (Bio‐Rad). For two‐dimensional electrophoresis and Western blotting, frozen spinal cords were homogenized in a lysis buffer (180 mM KCl, 5 mM MOPS, 2mM EDTA, 1 mM butylated hydroxytoluene and protease inhibitors), sonicated for 2 minutes at 4°C in an Ultrasonic processor UP50H and centrifuged for 5 minutes at 1000 × g; the supernatant was collected and centrifugated again (5 minutes, 1000 × g). After quantification, 1 mg of protein was precipitated with 20% trichloroacetic acid and the pellet was resuspended in 200 ml of denaturizing buffer (9 M urea, 4% CHAPS). Proteins of 100–200 mg were dissolved in isoelectric focusing buffer (9 M urea, 4% CHAPS, 1% bromophenol blue, 50 mM DTT and 0.5% ampholites). The solution was applied overnight to 3–11NL 18cm IPG strips (GE Healthcare Bio‐Sciences AB, Uppsala, Sweden) and isoelectric focusing migration was performed in a Bio‐Rad system. Strips were derivatized in a 0.2% 2,4‐dinitrophenylhydrazine solution in 2N Hydrochloric acid for 10 minutes and equilibrated with DTT and iodoacetamide. The equilibrated strips were loaded in a 10% SDS‐PAGE gel (20 × 20 cm), proteins were then transferred onto nitrocellulose membranes and detected with ECL Western blotting analysis system followed by exposure to CL‐XPosure Film (Thermo Scientific). Differentially oxidized spots were analyzed by MALDI‐reTOF MS.

### Protein identification

Protein identification was performed in the Proteomics Unit of the Vall d'Hebron Institut de Recerca (VHIR). Briefly, after excision from gel, proteins were reduced with 10 mM DTT and alkylated with 55 mM iodoacetamide. Enzymatic digestion was performed with trypsin following conventional procedures 21. After evaporation and re‐dissolution in methanol/water (1:2 v/v), 1% acetic acid digests were analyzed by matrix‐assisted laser desorption/ionization reflectron time‐of‐flight (MALDI‐reTOF MS). The MALDI‐reTOF MS analysis of the samples was performed using a Voyager DE‐PRO MALDI‐reTOF mass spectrometer (Applied Biosystems, Foster City, CA). Swiss‐Prot and GenBank data bases were used for the protein identification from the peptide mass fingerprinting from MALDI‐reTOF MS.

### PLP‐DM20 mRNA quantification in nerves and skin fibroblasts from PMD patients with a *PLP1* duplication

After written patient consent, peripheral nerve biopsies were obtained during orthopedic surgery for hip dislocation or scoliosis (Pr A. Tanguy, pediatric surgery department, Clermont‐Ferrand University Hospital). Samples were collected from 6 PMD patients with a *PLP1* duplication, aged from 3 to 17 years (mean = 8 +/– 5 years) and from 6 age matched control subjects with non‐neuromuscular cause of congenital dislocation of the hip or idiopathic scoliosis. Samples were immediately frozen in liquid nitrogen. Skin biopsies were obtained after written consent for 14 PMD patients with a *PLP1* duplication aged from 1 to 35 years (mean = 12 +/– 11 years) and from 10 age matched control subjects. For 5 patients, the skin biopsy was obtained concomitantly to the peripheral nerve biopsy.

Briefly, total RNAs were extracted using Trizol reagent (Invitrogen) and were reverse transcribed using oligodT primer and SuperScript^TM^ III reverse transcriptase Invitrogen) following the manufacturer's instructions. Quantitative PCR was performed on Light Cycler (Roche, Roche Diagnostics GmbH, Mannheim, Baden‐Württemberg, Germany) using the SybrGreen technology; quantification of PLP‐DM20 mRNA level was carried‐out regarding β2 microglobulin mRNA. For each patient, two independent reverse transcription reactions (RT1 and RT2) were performed and then, PCR quantification for each gene was done twice (values A and B) on both RT reactions. For further information, see the Supporting Information.

### Cell culture and treatments

Primary fibroblast cultures were established for each PMD patient with *PLP1* gene duplications from skin biopsies with written patient consent by the LeukoFrance Biobank (INSERM 1141 and Cytogenetic Department, CHU, Clermont Ferrand, France). Fibroblasts from four healthy age‐matched at the time of biopsy (10 months to 13‐year old) male subjects were used as controls (kindly provided by MT Zabot, Biotechnology Center, Hospices Civils de Lyon, Bron, France). The cultures were obtained in 25‐cm^2^ flasks with RPMI‐1640 medium (Invitrogen, Carlsbad, CA), supplemented with 4 mM glutamine (Invitrogen), 10% fetal bovine serum (Invitrogen), 100 U/mL penicillin‐100 mg/mL streptomycin (Invitrogen) and 2.5 mg/ml amphotericin B (ATGC, Marne La Valle´e, France) and maintained at 37°C in a 5% CO_2_ environment. Cells were then grown as previously described 22. Briefly, cells were cultured in DMEM (containing 10% fetal bovine serum, 100 U/ml penicillin and 100 mg streptomycin). To test the effects of the antioxidant NAC, PMD human fibroblasts were treated at 80% confluence in fresh growth medium for 24 h with NAC (100 μM) 41. To induce mitochondrial fission, human fibroblasts were exposed to serum depletion for 8 h 60 or treated for 1 h with CCCP (200 μM) 32.

### Analysis of mitochondria morphology

Control and PMD patient fibroblasts were fixed with 4% PFA and mitochondria were stained with the anti‐Tom20 antibody and nucleus with DAPI. Cells were imaged by a Leica TCS SL Laser scanning confocal spectral microscope (Leica Microsystems Heidelberg, GmbH, Mannheim, Germany) with a 63× oil immersion objective. To observe individual mitochondria, we acquired *z*‐stack images in series of eight slices per cell with thickness 0.4 μm per slice. Confocal images were processed using the NIH‐developed Image J software. Cells were thresholded to select mitochondria and binary (black and white) images were generated. Then, the perimeter, average size and the circularity of each mitochondria were quantified using the Analyze Particle function.

### Evaluation of intracellular radical oxygen species

Intracellular ROS levels were estimated using different ROS‐sensitive fluorescent probes and ROS levels were calculated as described 22. DCF and DHE were used to quantify intracellular ROS. MitoSOX™ Red was used to quantify mitochondrial ROS. Following incubation with 10 μM DCF for 30 minutes, 5 µM DHE for 30 minutes or 5 µM MitoSOX™ Red for 15 minutes, cells were washed with PBS 1X and lysated with 1% Triton. The fluorescence of stained cells was measured with a spectrofluorimeter. Wavelength filters used were: excitation wavelength 485 nm, emission wavelength 520 nm for DCF and excitation wavelength 530 nm, emission wavelength 590 nm for DHE and MitoSOX™ Red. Antimycin A was used as positive control.

### Inner mitochondrial membrane potential quantification by flow cytometry

Treated cells were washed with PBS 1X and incubated with 50nM of TMRE in pre‐warmed PBS 1X for 30 minutes at 37°C. Next, cells were trypsinized, centrifugated at 1000 × g for 5 minutes and resuspended in pre‐warmed PBS 1X. All samples were captured in a FACS Canto^TM^ recording 5000 cells for each condition and genotype tested. Histograms showing the percentage of depolarized cells were obtained after gating live cells. Data were analyzed with FlowJo Tree Star software.

### ATP measurements

ATP levels in fibroblasts, spinal cord and brain were measured by a chemiluminescence system using ATPlite1step (PerkinElmer, Waltham, Massachusetts), according to the manufacturer's protocol. ATP levels were normalized by total protein concentration.

### High resolution respirometry

Mitochondrial respiration in intact fibroblasts was evaluated using a high‐resolution respirometer (Oroboros, Oxygraph‐2k). Briefly, 1 × 10^6^ cells were resuspended in 2 ml of DMEM medium and added in the chamber at 37°C. After 20 minutes in the presence of constant stirring, the endogenous respiration (R′) was established. Following the stabilization of the R′, oxygen consumption was measured in the presence of 2 µg/ml oligomycin to obtain the oligomycin‐insensitive (leak) state (L′). Subsequently, the maximal electron transport system capacity (E′) of the mitochondria was achieved by titrating sequentially with repeated 1 µl doses from 1 mM carbonyl cyanide‐4‐(trifluoromethoxy)phenylhydrazone (FCCP) until a maximal response (final concentration 8 μM). Finally, non‐mitochondrial respiration or ROX was measured by the addition of 2.5 µM antimycin A. ROX values were used to correct the apparent respiratory fluxes (R = R′‐ROX; L = L′‐ROX; E = E′‐ROX). RCR represents the maximum electron transport capacity over the oligomycin‐insensitive respiration (E/L). OSR was calculated by subtracting the oligomycin‐insensitive respiration to the endogenous respiration (R‐L). MRC values were obtained from the ratio between the maximal electron transport chain capacity and the endogenous respiration 9, 58.

### RNA and DNA extraction

Total RNA was extracted using RNeasy Kit (Qiagen) according to the manufacturer's instructions. Total DNA was extracted using Gentra Puragene Tissue Kit (Qiagen) according to the manufacturer's instructions.

### Quantitative real‐time PCR

Gene expression and mtDNA levels were determined by Taqman real time PCR as previously described 48. Briefly, one microgram of RNA was transcribed into complementary DNA by SuperScript® II reverse transcription system (Invitrogen). Complementary DNA (0.1–0.2 ml) and 100 ng DNA were used to measure gene expression and mitochondrial DNA levels, respectively. TaqMan real‐time PCR was performed using the TaqMan Universal PCR master mix and the standardized primers for mouse *PGC‐1α* (Mm00447183), *NRF‐1* (Mm00447996), *NRF2* (Mm00477786), *TFAM* (Mm00447485), *RIP140* (Mm01343436), *GPX1* (Mm00656767), *Catalase* (Mm00437992), *SOD1* (Mm01700393), *SOD2* (Mm00449726), *DRP1* (Mm01342903), *Fis1* (Mm00481580), *OPA1* (Mm00453879), *MFN1* (00612599) and *MFN2* (00500120). To quantify DNA content, mouse *cytb* probe was used (Custom TaqMan Gene Expression Assays; Applied Biosystems). Quantification of mitochondrial DNA was referred to nuclear DNA as determined by the amplification of the intronless nuclear gene *CEBP* (Mm00514283). Expression of the genes of interest was normalized to that of the reference control mouse *RPLP0* (Mm01974474).

### Pyruvate kinase activity

PK activity was quantified by a spectrophotometrical method as previously described 23. Briefly, 15 μg of mitochondria‐free supernatant were added to a 0.2 ml of reaction buffer with 50mM Tris‐HCl pH 7.4, 100 mM potassium chloride, 20 mM magnesium chloride, 0.3 mM NADH, 4 mM ADP, 1 mM phosphoenolpyruvate and 5 units/ml of lactate dehydrogenase. NADH was recorded at 340 nm. All assays were performed in duplicate. Results were expressed as units per mg tissue.

### Aspartate aminotransferase activity

Aspartate aminotransferase activity was determined by a spectrophotometrical method as previously described 6. Briefly, in a microplate 160 μl of reaction buffer (90mM Tris‐HCl pH 6, 20 mM l‐aspartate, 150 μM pyridoxal 5′‐phosphate, 225 μM NADH, 950U/L lactate dehydrogenase and 750 U/L malate dehydrogenase were added to 20 μl of sample diluted at a 0.25 μg/μl in extraction buffer (0.32 M sucrose, 15 mM Tris‐HCl pH 7.4, 1 mM EDTA (ethylenediaminetetraacetic acid), 0.5 mM DTT, protease inhibitor cocktail and phosphatase inhibitor cocktail) and incubated 5 minutes at 37°C. Then, 20 μl of oxoglutarate 150 mM were added and finally NADH was spectrophotometrically recorded after 8 minutes at 340 nm in a microplate spectrophotometer (PowerWave Microplate Spectrophotometer, BioTek). All assays were performed in duplicate. Results were expressed as units per mg tissue.

### Glutathione reductase activity

Glutathione reductase activity was determined with a spectrophotometrical method (NWLSSTM Glutathione Reductase Activity microplate assay, NWK‐GR01, Northwest, Vancouver, Canada). Briefly, 90 μg of tissue in the same buffer as for aspartate aminotransferase activity were added to 100 μl of a reaction buffer containing glutathione disulfide and NADPH. NADPH was spectrophotometrically recorded at 340 nm in a microplate spectrophotometer (PowerWave Microplate Spectrophotometer, BioTek), 35 minutes after starting the reaction. All assays were performed in triplicate at room temperature. Results were expressed as U/mg tissue.

### Statistical analysis

After verifying normality (Shapiro‐Wilk test), statistical significance was assessed using the Student's *t*‐test whenever two groups were compared. When analyzing multiple groups, we used a one‐way ANOVA or two‐way ANOVA and Tukey's *post hoc* test to determine statistical significance except for PLP‐DM20 mRNA quantification for which a nonparametric Wilcoxon test was performed. Data are presented as the mean ± standard deviation (SD) (*, *P* < 0.05; **, *P* < 0.01; ***, *P* < 0.001).

## Results

### Spinal cord neuropathological analysis in PLP‐tg^66/66^ mice

**Figure 1:**
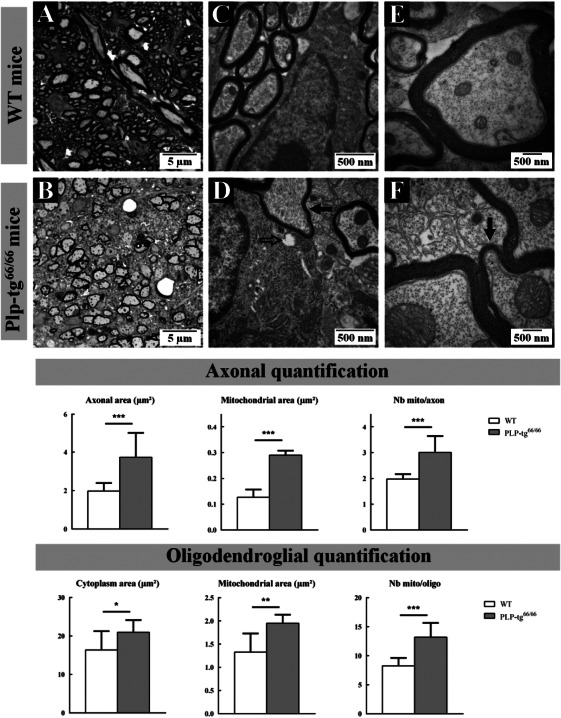
*Neuropathological analysis of the white matter cervical spinal cord of 6‐week‐old wild‐type and Plp1 overexpressing (PLP‐tg^66/66^) mice*. Seven hundred‐nanometer semithin epoxy embedded cervical spinal cord sections from WT (**A**) and PLP‐tg^66/66^ (**B**) mice stained with toluidine blue. Photographs taken at 100× length of ventral column white matter (**A**, **B**). Seventy‐nanometre ultrathin sections stained with uranyl acetate and lead citrate from WT (**C**, **E**) and PLP‐tg^66/66^ (**D**, **F**) obtained from the same block than semithin sections. Electron micrographs of sections from WT (**C**) and PLP‐tg^66/66^ (**D**) magnification ×25 000 and from WT (**E**) and PLP‐tg^66/66^ (**F**) magnification ×50 000. Large unmyelinated axons were very frequently observed in PLP‐tg^66/66^ mice (**B**, **D**). Of note, when myelin was preserved, the sheets exhibited normal compaction and periodicity, but reduced thickness (Black filled arrows; **D** and **F**). An increase in the density or size of the mitochondria was identified in PLP‐tg^66/66^ oligodendrocytes, with some of the mitochondria appearing degenerated (Black empty arrows; **D**). Quantifications made in 3 WT and 3 PLP‐tg^66/66^ confirmed the increase in size of both axon and oligodendrocyte cytoplasm in PLP‐tg^66/66^ as well as the increase in mitochondria size and number. Statistical analysis was performed by two‐way ANOVA; *P < 0.05, **P < 0.01; ***P < 0.001.

We focused on neuropathological analysis of spinal cords from 6‐week‐old PLP‐tg^66/66^ mice because of the key role of long spinal tracts in the progression of PMD neurological symptoms. Toluidine blue staining and electron microscopy revealed a greater number of unmyelinated axons in the white matter of PLP‐tg^66/66^ mice compared to WT mice. Axons of all calibers were hypomyelinated (Figure 1A,B). Preserved myelin sheets exhibited normal compaction and periodicity, but reduced thickness (Figure 1C–F). These data are in agreement with previous histological studies 33, 61. The vast majority of axons exhibited an increased diameter associated with an increase in mitochondria number and size (Figure 1). Furthermore, in oligodendrocytes the surface of the cytoplasm occupied by mitochondria is markedly increased, with some mitochondria appearing degenerated (Figure 1D).

**Figure 2:**
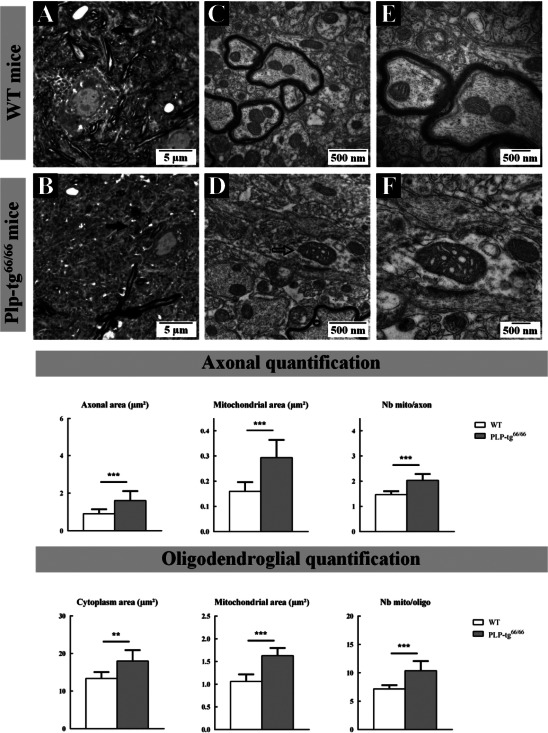
*Neuropathological analysis of the gray matter cervical spinal cord of 6‐week‐old wild‐type and Plp1 overexpressing (PLP‐tg^66/66^) mice*. Seven hundred‐nanometre semithin epoxy embedded cervical spinal cord sections from WT (**A**) and PLP‐tg^66/66^ (**B**) mice stained with toluidine blue. Photographs taken at 100× length of gray matter intermediate zone (**A**, **B**). Seventy‐nanometre ultrathin sections stained with uranyl acetate and lead citrate from WT (**C**, **E**) and PLP‐tg^66/66^ (**D**, **F**) obtained from the same block than semithin sections. Electron micrographs of sections from WT (**C**) and PLP‐tg^66/66^ (**D**) magnification ×25 000 and from WT (**E**) and PLP‐tg^66/66^ (**F**) magnification ×50 000. Numerous bundles of small axons well oriented in the rostrocaudal direction and markedly myelinated were observed in WT mice; these axons were unmyelinated in PLP‐tg^66/66^ mice (Black filled arrows; **A** and **B**). The electron microscopy analysis also revealed a dramatic increase in size of some mitochondria in the gray matter of PLP‐tg^66/66^ mice, many exhibiting also abnormal cristae morphology (Black empty arrows; **D**). Quantifications made in 3 WT and 3 PLP‐tg^66/66^ confirmed the increase in size of both axon and oligodendrocyte cytoplasm in PLP‐tg^66/66^ as well as the increase in mitochondria size and number. Statistical analysis was performed by two‐way ANOVA; *P < 0.05, **P < 0.01; ***P < 0.001.

Gray matter of spinal cords of 6‐week‐old PLP‐tg^66/66^ also demonstrated abnormalities. Notably, the numerous bundles of small myelinated axons observed in WT mice were severely hypomyelinated in PLP‐tg^66/66^ (Figure 2A,B). Similar to the white matter, we found a significant increase in the number and size of mitochondria in the gray matter of PLP‐tg^66/66^ (Figure 2C–F). This increase is observed both in axons and the cytoplasm of oligodendrocytes (Figure 2). These observations in the spinal cord are in accordance with results reported in the brain 29.

Finally, electron microscopy experiments showed abnormal mitochondrial morphology and damaged mitochondrial structure in axons and oligodendrocytes in white and gray matter from cervical spinal cord of 6‐week‐old Plp1 over expressing (PLP‐tg^66/66^) mice. Bigger mitochondria with swollen/altered cristae were detected in axons and oligodendrocytes from Plp1 over expressing (PLP‐tg^66/66^) mice (Figures 1E,F and 2E,F).

### Mitochondrial DNA and protein levels are increased in PLP‐tg^66/66^ spinal cords

**Figure 3:**
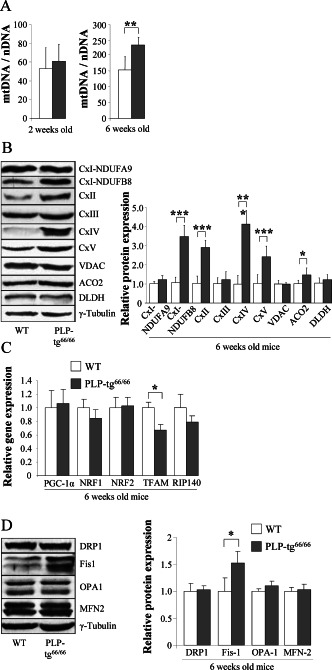
*MtDNA and protein levels are increased in PLP‐tg66/66 spinal cords at 6 weeks of age*. mtDNA content in 2‐ and 6‐weeks old mice is expressed as the ratio of mtDNA (cytb) to nuclear DNA (CEBP) (**A**). CxI subunits NDUFA9 and NDUFB8, CxII, CxIII, CxIV, CxV, VDAC, ACO2 and DLDH protein expression in 6‐week‐old mice (**B**). Relative gene expression of *Pgc*‐*1α*, *Tfam*, *Nrf1*, *Nrf2* and *Rip*‐*140* in 6‐week‐old mice (**C**). Relative protein expression of DRP1, Fis1, OPA1 and MFN2 in 6‐week‐old mice spinal cord (**D**). Representative blots are shown. The protein level is expressed as a fold increase of the control and in reference to γ‐tubulin as a loading marker. Two‐week‐old mice n = 7/genotype; 6‐week‐old mice, WT n = 6, PLP‐tg^66/66^ n = 7. CTL fibroblasts n = 4; *PLP1*‐duplicated PMD fibroblasts n = 4. Values are expressed as the mean ± SD. Statistical analysis was performed using Student's *t*‐test; *P < 0.05, **P < 0.01, ***P < 0.001.

To further investigate the role of mitochondrial dysfunction in the pathogenesis observed in *Plp1* mutants, we first analyzed mitochondrial number and biogenesis by determining the mitochondrial DNA/nuclear DNA ratio (mtDNA/nDNA) and the protein levels of different mitochondrial proteins in whole extracts of PLP‐tg^66/66^ mice spinal cords. At 6 weeks of age, mtDNA copy number was significantly increased (Figure 3A), together with the protein levels for the majority of the mitochondrial proteins analyzed, most of which were integrated in the OXPHOS system (Figure 3B). Interestingly, VDAC, a mitochondrial porin whose expression is correlated with mitochondrial abundance 44, was not altered (Figure 3B), suggesting that the increase in OXPHOS‐related proteins is not an indirect consequence of the increase in mitochondrial number. This was a progressive, accumulative phenomenon, as the mtDNA/nDNA ratio was still normal at 2 weeks of age, before the onset of symptoms (Figure 3A).

The best studied molecular mechanism mediating increased mitochondrial biogenesis involves PGC1α, a co‐transcriptional regulation factor that controls a cascade of events inducing mitochondrial gene transcription, including NRF‐1 and −2 which in turn activate mitochondrial TFAM to drive transcription and replication of mitochondrial DNA 83. To investigate whether this signaling pathway was involved in the increased mitochondrial numbers observed in PLP‐tg^66/66^ mice spinal cords, we measured mRNA levels of *PGC1α*, *NRF1*, *NRF2*, *TFAM* and also *RIP‐140*, the latter being a key factor in the repression of metabolic gene expression and mitochondrial function. We found a significant down‐regulation of *TFAM* only, while all the other genes showed normal expression levels (Figure 3C) indicating that an alternative mechanism, such as impaired mitochondrial dynamics may be at play accounting for the increased mitochondrial number and size observed by electron microscopy in the spinal cords of PLP‐tg^66/66^ mice. Mitochondrial dynamics, or the delicate balance between fission and fusion, may contribute to the pathogenesis of diseases not classically considered to be of mitochondrial origin, and it may contribute to the observed mitochondrial size, number and functionality 4. Some perturbations of this process have been shown to affect mitochondrial morphology, provoking a rapid accumulation of deleterious mtDNA mutations and loss of mitochondrial functions in mouse and human cell culture 13, 45, 73. In some neurodegenerative disorders as Alzheimer Disease (AD), an altered balance in mitochondrial fission and fusion leading to mitochondrial and neuronal dysfunction has been revealed in brain and fibroblasts, with different protein expression level pattern in each tissue 78, 79. To investigate whether the fission and fusion balance was altered in the PMD model, we measured levels of the main proteins involved in this process: dynamin related protein 1 (DRP1), fission protein 1 (Fis1), optic atrophy 1 protein (OPA1), mitofusin‐1 (MFN1) and mitofusin‐2 (MFN2), in the spinal cords of PLP‐tg^66/66^ mice. Unexpectedly, we found a significant induction of Fis1 protein level while mitochondria presented increased quantity and size in the spinal cords of PLP‐tg^66/66^ mice (Figure 3D). These data suggest malfunction in mitochondrial dynamics, whereby fission is initiated with proper Fis1 induction, but fails to engage the subsequent steps, resulting in fission arrest and thus, increased mitochondrial size. Similar findings of incomplete fission are found in aging‐related processes and Alzheimer disease 49, 86. The situation in brain and human fibroblasts is different, with normal mtDNA/nDNA ratio and mitochondrial protein expression (Supporting Information Fig. S1A, B, C). However, both *PGC1α* and *RIP‐140* were upregulated at 6‐weeks in the mutant brains, most likely counteracting each other and accounting for the unchanged mtDNA/nDNA ratio (Supporting Information Fig. S1D). Fission and fusion proteins were not dysregulated (Supporting Information Fig. S1D). These results suggest more severe tissue damage in the spinal cord than in the brain, with compensatory changes in expression of the mitochondrial biogenesis regulators *PGC1α* and *RIP140* observed only in the latter.

### Oxidative stress and damage to proteins in PLP‐tg^66/66^ spinal cords

**Figure 4:**
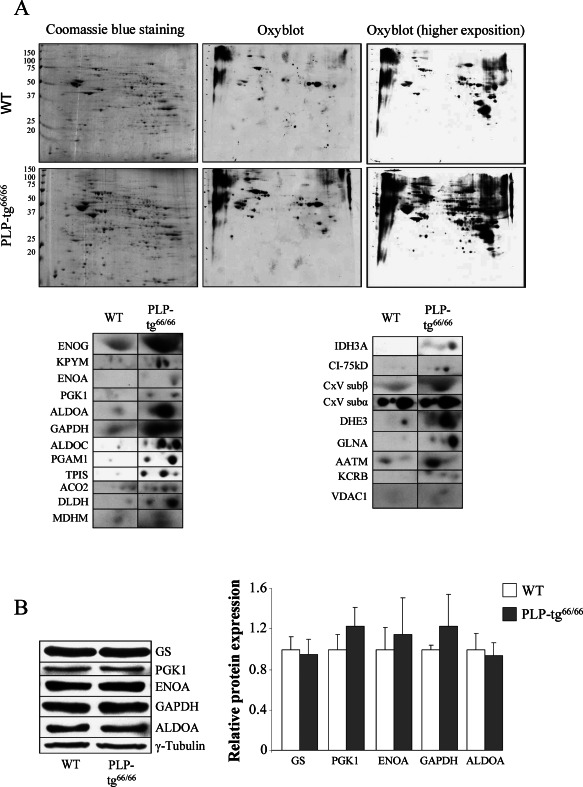
*Twenty one more markedly oxidized proteins identified in 6‐week‐old PLP‐tg^66/66^ mice in the spinal cord*. **A.** Redox proteomics experiments were performed in 6‐week‐old PLP‐tg^66/66^ and their matched WT mice. Western blot with an anti‐ dinitrophenylhydrazine antibody at different exposure times to chemiluminescent reactive was performed to detect oxidized proteins (n = 3/genotype). **B.** All non‐mitochondrial oxidized proteins tested by Western blot in the spinal cord are normally expressed. Relative protein level expressed as a percentage of control and in reference to γ‐tubulin as a loading marker (n = 6/genotype). Values are expressed as the mean ± SD. Student's *t*‐test was used for statistical analysis; *P* < 0.05, **P < 0.01, ***P < 0.001.

Impaired mitochondrial function generally leads to oxidative stress in neurodegenerative diseases 39. Thus, we next evaluated the presence of oxidative damage in whole tissue extracts of PLP‐tg^66/66^ spinal cords. We used a well‐established, two‐dimensional‐based redox proteomics approach as previously described 23, 42, 43. We detected increased oxidation in 21 proteins linked to energetic/mitochondrial/glutamine metabolism (Figure 4A, Table 1) in the PLP‐tg^66/66^ mutants, at 6 weeks of age; among them, nine proteins involved in glycolysis, several OXPHOS components including two subunits of the ATP synthase, and CK‐BB, evidencing an intertwining of redox and bioenergetic homeostasis. Posttranslational modifications of CK‐BB are implicated in aging and other neurological disorders such as Alzheimer's disease, multiple sclerosis or Huntington's disease 11, 12, 14, 56, 57.

**Table 1:** Identification of carbonylated proteins by peptide mass fingerprint and/or TOF‐TOF PSD. Twenty‐one proteins related to energetic/mitochondrial/glutamate metabolism were identified.

Identified protein	Access/short name	pI	Mw (Da)	MASCOT Score
**Glycolysis**				
Gamma‐enolase	ENOG	4.99	47609	213
Pyruvate kinase isozymes M1/M2	KPYM	7.18	58378	107
Alpha‐enolase	ENOA	6.37	47453	193
Phosphoglycerate kinase 1	PGK1	8.02	44921	124
Fructose‐bisphosphate aldolase A	ALDOA	8.31	39787	211
Glyceraldehyde‐3‐phosphate dehydrogenase	G3P, GAPDH	8.44	36072	151
Fructose‐bisphosphate aldolase C	ALDOC	6.67	39769	229
Phosphoglycerate mutase 1	PGAM1	6.67	28928	109
Triosephosphate isomerase	TPIS	6.9	27038	288
**Citric acid cycle**				
Aconitase hydratase, mitochondrial	ACO2	7.87	85372	62
Malate dehydrogenase, mitochondrial	MDHM	8.93	36045	142
Isocitrate dehydrogenase [NAD] subunit alpha, mitochondrial	IDH3A	6.27	40069	185
Dihydrolipoyl dehydrogenase, mitochondrial	DLDH	7.99	54751	120
**Electron transport chain**				
NADH‐ubiquinone oxidoreductase 75 kDa subunit, mitochondrial	NDUS1, CxI sub‐75kD	5.51	80724	199
ATP synthase subunit alpha, mitochondrial	ATPA, CxV subα	9.22	59830	138
ATP synthase subunit beta, mitochondrial	ATPB, CxV subβ	5.19	56265	167
**Glutamine metabolism**				
Glutamate dehydrogenase 1, mitochondrial	DHE3	8.05	61640	252
Glutamine synthetase	GLNA	6.64	42834	149
Aspartate aminotransferase, mitochondrial	AATM, GOT	9.13	47780	103
**Other**				
Creatine kinase B‐type	KCRB	5.4	42971	164
Voltage‐dependent anion‐selective channel protein 1	VDAC1	8.55	32502	208

This increase in oxidation was not because of higher amount of protein in the sample, or higher protein expression of non‐mitochondrial proteins, as shown by Western blots (Figure 4B). Protein oxidation was not observed at 2 weeks of age (data not shown), indicating a phenomenon worsening with time and correlating with the onset of neurological damage. Thus, in PLP‐tg^66/66^ mice, mitochondrial impairment is linked to an increase in oxidative stress.

### Bioenergetic failure in PLP‐tg^66/66^ spinal cords

**Figure 5:**
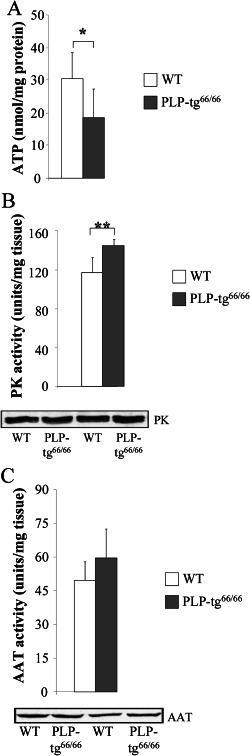
*Plp1 overexpression disturbs energetic metabolism in the spinal cord from 6‐week‐old PLP‐tg^66/66^ mice*. ATP levels are decreased (**A**) and PK is increased (**B**) in PLP‐tg^66/66^. AAT is not significantly increased (**C**). Activity is expressed as units/mg tissue. WT (n = 6) and PLP‐tg^66/66^ mice (n = 7) were used for the study. Values are expressed as the mean ± SD. Student's *t*‐test was used for statistical analysis; ***P < 0.001, **P < 0.01, *P < 0.05.

We next studied the possible consequences of protein oxidation on energetic homeostasis by quantifying ATP content in PLP‐tg^66/66^ mice spinal cords at 6 weeks of age. We found that ATP was reduced by 40% in these samples (Figure 5A), a direct indication of bioenergetic failure. We also measured the activity of pyruvate kinase (PK) to test the effect of decreased ATP levels on glycolysis. PK activity was significantly increased in PLP‐tg^66/66^ mice spinal cords (Figure 5B), suggesting an unsuccessful attempt to offset the mitochondrial ATP deficit by increasing glycolytic activity.

In the brain of 6‐week‐old PLP‐tg^66/66^ mice, both ATP content and PK activity were increased (Supporting Information Fig. S2A and B), suggesting tissue specificity of the metabolic impairment, a compensatory mechanism that takes place in the brain or an earlier stage of the pathogenesis cascade in the brain.

### Glutamate metabolism in PLP‐tg^66/66^ spinal cords

Glutamine is not simply a precursor to neuronal glutamate but a potential fuel which, like glucose, supports neuronal energy requirements 15, 19, 85. Three main enzymes are involved in the glutamate‐glutamine cycle: glutamine synthetase, glutamate dehydrogenase 1 and aspartate aminotransferase (AAT), which were found carbonylated by redox proteomics (Table 1). We thus determined the total activity of AAT, the rate‐limiting enzyme involved in glutamate oxidation. We found no significant difference in AAT activity (Figure 5C) in PLP‐tg^66/66^ mice spinal cord extracts suggesting that, despite oxidation, the activity of the enzyme was not affected, possibly because of a feedback mechanism to maintain the pool of tricarboxylic acid metabolites and thus, energy production. In the brain in contrast, AAT activity was significantly increased (Supporting Information Fig. S2C). This reinforces the notion of an adaptive brain‐specific response of the bioenergetic metabolic network to counteract, at least in part, the bioenergetic challenges presented by *PLP1* overexpression.

### Adaptive enzymatic antioxidant defense in PLP‐tg^66/66^ spinal cords

**Figure 6:**
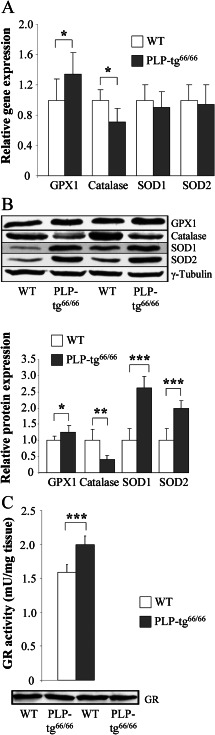
*Antioxidant defense is altered in spinal cords from 6‐week‐old PLP‐tg^66/66^ mice*. Antioxidant enzyme RNA (**A**) and protein (**B**) levels and GR activity (**C**) were determined in spinal cords from 6‐week‐old WT and PLP‐tg^66/66^mice (n = 7/genotype). RNA was quantified by TaqMan real time PCR. Relative protein level is expressed as a percentage of control, and referred to γ‐tubulin as loading marker. Glutathione reductase activity is expressed as units/mg tissue. Values are expressed as the mean ± SD. Student's *t*‐test was used for statistical analysis; *P < 0.05, **P < 0.01, ***P < 0.001.

Oxidative stress is caused by an imbalance between continuous ROS production and the enzymatic antioxidant shield of the cell. To explore whether a malfunctioning antioxidant response was involved in the oxidative damage observed in PLP‐tg^66/66^ mice spinal cord at 6 weeks, we determined the RNA and protein levels of the main detoxifying enzymes involved in ROS scavenging: glutathione peroxidase 1 (GPX1), catalase, SOD1 and SOD2. In the mutant spinal cords, we found an induction of *GPX1* at the mRNA and protein levels, reflecting a physiological response to increased free radicals (Figure 6A,B). Both cytosolic (SOD1) and mitochondrial (SOD2) SOD were unchanged at the mRNA level, but more than twofold induced at the protein level in PLP‐tg^66/66^ mice (Figure 6A,B). However, catalase was significantly down regulated (Figure 6A,B), suggesting a suboptimal antioxidant response against hydrogen peroxide excess. No significant variation of the above‐cited enzymes was found in PLP‐tg^66/66^ mice brain at the same age (Supporting Information Fig. S3A, B).

In its reduced state, glutathione is the main antioxidant of the cell, preventing damage to important cellular components caused by ROS. As a result of redox homeostasis, glutathione is converted to its oxidized form, glutathione disulfide. Once oxidized, glutathione can be reduced back by glutathione reductase (GR), the rate‐limiting enzyme of the antioxidant process. We thus measured GR activity in PLP‐tg^66/66^ mice spinal cord at 6 weeks, and found a significant increase in activity, not related to an increase in protein expression (Figure 6C). This may reflect a physiological response to the increased ROS, as was seen with increased GPX1 and SOD expression. GR activity was also increased in the brain, suggesting an early and robust step in the adaptive response to oxidative stress in the CNS (Supporting Information Fig. S3C).

### PLP‐DM20 mRNA quantification in nerves and skin fibroblasts from PMD patients with a *PLP1* duplication

**Figure 7:**
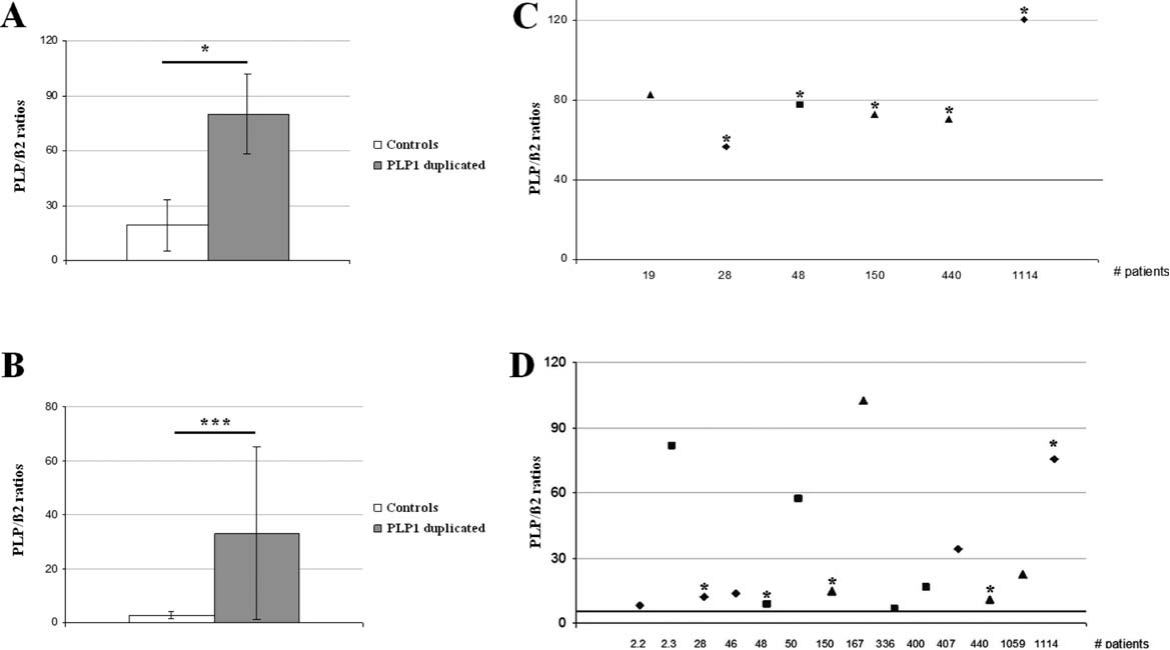
*PLP‐DM20 mRNA quantification in nerves and skin fibroblasts from PMD patients with a PLP1 duplication*. Histograms represent the mean ratio obtained for control and *PLP1* duplicated patient nerves (**A**) and fibroblasts (**B**). Graphs represent individual data obtained for each *PLP1* duplicated patient nerves (**B**) and fibroblasts (**D**) as well as a pathological threshold value (bold line) determined by a ROC curve using ratios from patients and from controls. Severity of the disease is mentioned as: form 1 by triangle, form 2 by a square and form 3 by a rhomb. *mentioned the patients with results obtained in both the fibroblasts and the peripheral nerve (**C**, **D**). Statistical analysis was done by non‐parametric Wilcoxon test (**A**, **B**); *P < 0.05, **P < 0.01; ***P < 0.001.

We next determined whether the altered mitochondrial function and redox homeostasis observed in the mouse model was relevant for the human disease by analyzing primary fibroblasts derived from four *PLP1*‐duplicated PMD patients. In a first step, we evaluated if PLP overexpression was observed in nerves and fibroblasts from our PMD patients. The analysis of nerves was used to validate our quantification technique on a tissue with higher expression of PLP‐DM20 mRNAs. The mean of PLP/β2 ratios was 19 ± 13.8 in the control group (minimum 0.36, maximum 39.9) and 80.05 ± 21.7 (minimum 56.5, maximum 120.5) in the PMD group, demonstrating a significant PLP‐DM20 mRNA overexpression in the nerves from PMD patients with a PLP1 duplication. Using the obtained ratios, a pathological threshold of 39.9 was determined by drawing a ROC curve (Figure 7A,B).

PLP‐DM20 mRNAs quantification was then performed on fibroblasts from 14 PMD patients with a PLP1 duplication (including the 5 PMD patients for whom nerve analysis was carried out and 9 other) in comparison to fibroblasts from 10 control subjects. The mean of PLP/β2 ratios was 2.69 ± 1.31 (minimum 0.85, maximum 5.25) in the control group and 33.15 ± 32.18 (minimum 6.54, maximum 102.33) in the PMD group demonstrating a significant PLP‐DM20 mRNA overexpression in the fibroblasts from PMD patients with PLP1 duplications. Using the obtained ratios, a pathological threshold of 5.25 was determined by drawing a ROC curve (Figure 7C,D). The fibroblasts from the four PMD patients involved in the rest of the study were derived from the PMD Form 1 patients (triangle) analyzed in this experiment.

### Oxidative stress and mitochondria malfunction in human PMD fibroblasts are prevented by NAC

**Figure 8:**
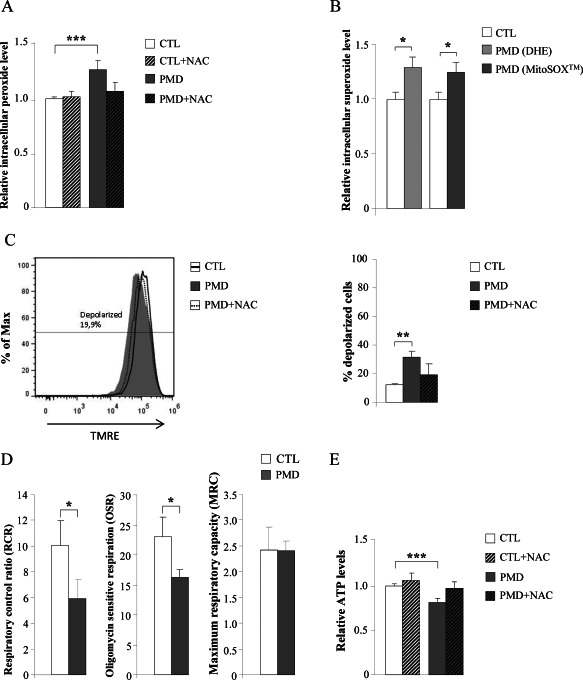
*Mitochondrial dysfunction in human PMD fibroblasts is prevented by NAC*. Intracellular peroxide levels (DCF probe) (**A**); Intracellular (DHE probe) and mitochondrial (MitoSOX probe) superoxide levels (**B**); Inner mitochondrial membrane potential (**C**); mitochondrial respiration (**D**), and ATP content (**E**) were quantified in human control and PMD fibroblasts with or without NAC treatment (n = 4/genotype). In **C**, the numeric value above the line corresponds to the percentage of depolarized cells in Pelizaeus‐Merzbacher disease fibroblasts after treatment with NAC (100 μM) during 24 h. DCF = dichlorofluorescein, DHE = dihydroethidium, TMRE = tetramethylrhodamine ethyl ester, CTL = control, PMD = Pelizaeus‐Merzbacher disease. Values are expressed as the mean ± SD. Statistical analysis was performed by one‐way ANOVA and Tukey's HSD *post hoc* (**A**, **C** and **E**) and Student's *t*‐test (**B** and **D**); *P < 0.05, **P < 0.01; ***P < 0.001.

We next quantified intracellular ROS fluorimetrically using the DCF probe, which measures intracellular peroxides 52, 77. At baseline, there were significantly higher ROS levels in fibroblasts of PMD patients compared to age matched controls (Figure 8A). We further investigated whether mitochondria could be the main cellular origin of these free radicals using the fluorescent dyes DHE and MitoSOX^TM^ Red (DHE covalently bonded to hexyl triphenylphosphonium cation), which measure intracellular and mitochondrial ROS levels, respectively 50. The ROS levels detected in human PMD fibroblasts using either DHE or MitoSOX^TM^ Red probes were similar (Figure 8B), indicating the mitochondria compartment as the principal source of ROS in these *PLP1* overexpressing fibroblasts.

Under the same experimental conditions, PMD fibroblasts displayed a partially depolarized inner mitochondrial membrane (Figure 8C). In addition, high‐resolution respirometry assays revealed a decreased respiratory control ratio (RCR), the single most useful test of mitochondrial function in cell populations 9, confirming impaired mitochondrial respiration in PMD fibroblasts. Further analysis showed that PMD fibroblasts had decreased oligomycin sensitive respiration (OSR), while the maximum respiratory capacity (MRC) remained unaffected (Figure 8D), suggesting that impaired activity of the mitochondrial H^+^‐ATP synthase (complex V of the OXPHOS system) could underlie the mitochondrial dysfunction in PMD fibroblasts rather than a defective electron transport. These data also correlated with significantly lower levels of ATP (Figure 8E). Interestingly, ROS production, mitochondrial depolarization and lower ATP levels were rescued using the antioxidant N‐acetylcysteine (NAC) (Figure 8A,C,E), indicating that oxidative stress may be the main culprit for the observed collapse of mitochondrial function.

### Impaired mitochondrial dynamics of PMD fibroblasts subjected to fission stimuli

The above data support that mitochondrial fitness is impaired in PMD. Potential mechanisms for this include an increase in the proportion of defective/damaged mitochondria as a consequence of impaired mitochondrial dynamics and quality control.

**Figure 9:**
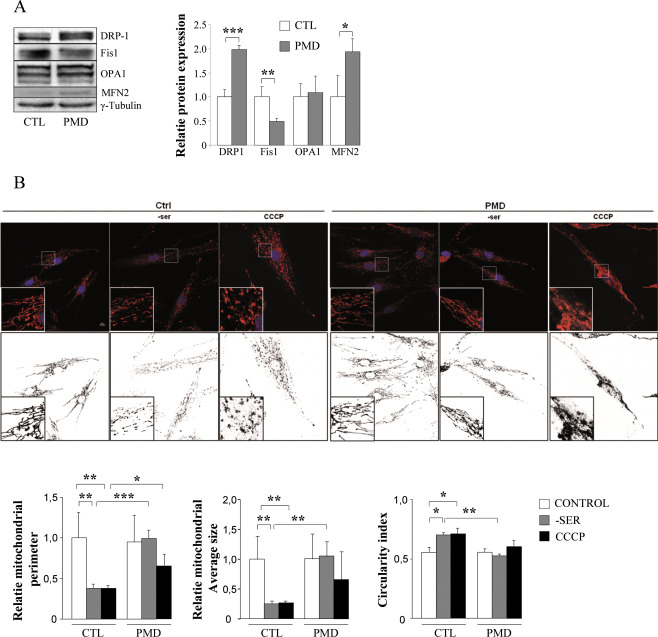
*Disturbed mitochondrial dynamics in human PMD fibroblasts on fission stimuli*. Relative protein expression of DRP1, Fis1, OPA1 and MFN2 in human Control and *PLP1*‐duplicated PMD fibroblasts (**A**). Confocal microscopy of stained Control and PMD fibroblasts with anti‐Tom20 antibody and DAPI (nuclei) following exposure to serum depletion for 8 h or CCCP (200 μM) for 1 h (**B**). Lower panels show enlarged areas of the white boxes in the above panels. Scale bar: 10 uM. Digital images were subjected to a convolve filter through the National Institutes of Health‐developed IMAGEJ software to isolate and equalize fluorescent pixels in the image. After thresholding, individual particles (mitochondria) were analyzed for diameter, average size and circularity. Lower panels show Software processed images (Black and white). A total of 20 cells (n = 3 Ctrl and n = 3 PMD) were examined for each condition. Statistical analysis was performed by one‐way ANOVA and Tukey's HSD *post hoc*; *P < 0.05, **P < 0.01.

We chose to verify this hypothesis in the human PMD fibroblasts used above. We first performed immunoblots against the fusion/fission machinery components: DRP1, Fis1, OPA1, MFN1 and MFN2. We found a significant increase of DRP1 and MNF2 protein levels while Fis1 level was decreased, suggesting a general dysregulation of the fission and fusion process (Figure 9A).

Then, control and PMD patient fibroblasts were stained with Tom20 antibody and mitochondria were visualized by fluorescence microscopy. Mitochondrial morphology was scored and representative confocal images of control and PMD patient fibroblasts are shown (Figure 9B). At basal level, no differences in mitochondrial morphology or distribution were observed between control and PMD patient fibroblasts. Indeed, PMD patient fibroblasts presented a normal, tubular and well distributed mitochondrial network (Figure 9B).

To induce fission and subsequent mitochondrial fragmentation, cells were exposed to serum depletion for 8 h as described by Rambold *et al* 60 or to the mitochondrial uncoupler CCCP for 1 h 32. Confocal imaging indicated extensive mitochondrial fragmentation after serum depletion in the control fibroblasts relative to PMD patient's fibroblasts which presented an unfragmented tubular network (Figure 9B). In a similar manner, extensive mitochondrial fragmentation evidenced by small, round or dot‐like staining patterns was induced in control fibroblasts treated with CCCP. In contrast, we observed a more condensed, less fragmented mitochondrial network in PMD patient's fibroblasts treated with CCCP. The degree of mitochondria fragmentation was analyzed by comparing the perimeter, average size and circularity of the mitochondria between the basal level and fission stimuli conditions. On serum depletion or CCCP treatment, we found an altered fission capacity of mitochondria in the fibroblasts from PMD patient fibroblasts in comparison with control fibroblasts.

## Discussion

### Oxidative stress and mitochondria malfunction in spinal cords of PMD mice

The PLP‐tg^66/66^ model is the most valuable mouse model of classical PMD because the clinical symptoms (early motor defects associated with subsequent neurodegeneration) and neuropathological presentation (severe and early hypomyelination) are very similar to the human disease. While mitochondrial ultrastructural defects were already described in the brains of these mice 3, 29, the results we present here reinforce and expand on these findings by providing a broader molecular base for these observations and uncovering the functional consequences relevant for disease pathology.

### The PMD brain exhibits a less severe or an earlier stage of the disease

Many differences were observed between the spinal cord and brain of PMD mice. We first showed normal mtDNA/nDNA ratio and mitochondrial protein expression levels in mutant brains compared to WT brains at 2‐ and 6‐weeks of age, even though both the antagonistic biogenesis regulators PGC1α and RIP140 were induced at 6‐weeks in the mutant brains. These data suggest less severe tissue damage in the brain, perhaps because of compensatory changes in mitochondrial regulatory pathways. An increased ATP content associated with increased PK and AAT activity at 6 weeks of age suggests tissue specificity of the metabolic impairment, or an earlier stage of the disorder in the brain. Finally, while no significant variation of the main ROS scavenging enzymes was found in the *Plp1* overexpressing brain, GR activity was increased, as also seen in the spinal cord. Together these data argue that there are more robust adaptive processes in the brain than in the spinal cord, or that the disease is less advanced at this time point in the brain.

Different lines of evidence have suggested that the toxicity of *PLP1* overexpression is only observed in myelinating oligodendrocytes: (i) the autopsy of a human fetus with a *PLP1* duplication showed normal development of oligodendrocyte precursors at the gestational age of 21 weeks 67; (ii) in classical PMD, the first symptoms are not observed at birth, and progress in the psychomotor development is clearly observed during the first years of life, identically to patients with cerebral palsy but with a subsequent neurodegeneration with an ascending progression when the active phase of myelination/synaptogenesis is finished. Myelination begins first in the spinal cord and subsequently progresses in the brain 5, suggesting a later involvement of the brain compared to the spinal cord in the physiopathology of the disease. Hence, it is conceivable that the spinal cord exhibits a more severe stage of the disease earlier than brain (for this study at 6 weeks of age). However, additional data may be necessary to discriminate between these two hypotheses.

### Are hypomyelination and its resulting increase in axonal energy requirements, the cause of the energetic crisis and excess of ROS production?

The involvement of ROS and oxidative stress has long been suspected as culprit in the pathogenesis of primary myelin disease, with possible involvement of altered mitochondrial function and distribution 51, 68. Several studies have reported an increase in mitochondrial densities in dysmyelinated mice 2, 28 and demyelinated lesions of the human brain 44, 81. Other reports have linked demyelination with an increase in the size of axonal stationary mitochondria and in the transport velocity of motile mitochondria, affecting both the distribution and the transport of axonal mitochondria 34. According to the authors, these modifications sustain the increase in energy demands of nerve conduction because of Na^+^ channel redistribution along the demyelinated axolemma. This is supported by an increase in *ATF3* expression, a stress response gene that facilitates axonal regeneration and survival 66. While no data are available for the severe hypomyelinated PLP‐tg^66/66^ mice, there was a wide redistribution of Na^2+^ channels along the axolemma in a mouse model expressing lower levels of *Plp1* (only 3–4 copies per genome) 74, 75. Thus, it is likely that the increase in the density of mitochondria observed in the PLP‐tg^66/66^ model parallels the increased energy demands of nerve conduction. It is possible that in chronically hypomyelinated axons, the adaptive mechanisms designed to maintain conduction may paradoxically lead to a vicious circle: when mitochondria are forced to operate at full capacity to provide enough energy to maintain conduction, the defective organelles may start generating more ROS. Thus, the increased energy requirement caused by hypomyelination could contribute to excessive ROS production and to oxidation of key enzymes of bioenergetic homeostasis, which aggravates the problem. This vicious circle could explain the severe growth failure observed in the PLP‐tg^66/66^ mice after 1 month of age.

Unlike other PMD point mutations where endoplasmic reticulum stress has been described as a pivotal physiopathogenic factor in the disease 40, 55, 69, an additional explanation for the excess of ROS is the mislocalization of PLP to mitochondrial membranes, as previously described 3, 29, where it may interfere with OXPHOS. Interference with OXPHOS is thought to produce electron leakage and free radicals in a number of classical mitochondrial conditions, and also in diseases with secondary mitochondrial involvement such as X‐linked adrenoleukodystrophy 43.

### The interdependence between oxidative stress and mitochondria malfunction is also observed in PMD patient's fibroblast, and circumvented with antioxidant treatment

Intriguingly, the interdependence that we observed between oxidative stress and mitochondrial malfunction was not restricted to the animal model, or to myelinating tissues. We interrogated the redox and bioenergetic homeostasis in patients' primary fibroblasts and identified: (i) mitochondria as the main source of ROS, (ii) increased numbers of depolarized mitochondria and lower levels of ATP and (iii) a decrease in the RCR and OSR. The antioxidant NAC restored bioenergetic balance, underscoring the interdependence of redox and bioenergetic homeostasis and suggesting that oxidative stress is the main factor responsible for the observed mitochondrial impairment, at least in fibroblasts. In a typical vicious circle scenario, malfunctioning partially depolarizes mitochondria generating ROS by electron leakage, which may oxidize its own OXPHOS and Krebs cycle machinery, and thus exacerbate ROS generation and bioenergetic deficits until eventual cellular collapse, as it happens in X‐ALD 43.

Bioenergetic failure has also been suspected in human PMD notably because of a slowed growth curve usually observed in patients after 4–6 years of age and worsening with the neurodegeneration. However, this energetic deficit has hitherto been little studied, notably because of the lack of post‐mortem tissue. Hence, the correlative data obtained in our study in the *bona fide* animal model, PLP‐tg^66/66^ mice, and in the fibroblasts of PMD patients with *PLP1* duplications, is an important step in understanding the cellular role of bioenergetic failure in this disease.

Another factor that may compromise mitochondria viability and contribute to cell demise is the presence of inflammatory mediators. In mice with experimental EAE (autoimmune encephalitis), an animal model for multiple sclerosis, inflammation causes the release of ROS, which attack mitochondria, producing swelling and fragmentation of its network, well before axonal degeneration occurs 53. Secondary neuroinflammation in *PLP1* transgenic mice has been proposed to contribute to axonal degeneration 17, 31. In a comparable model of *Plp1* overexpression, microglial activation and up‐regulation of pro‐inflammatory cytokines has been described in the brain as an early event before the start of myelination 76, which suggests that a situation detrimental to mitochondria may occur early in the pathogenesis cascade.

It follows that anti‐inflammatory antioxidant drugs with mitochondria protective properties could be of interest for PMD. For instance, pioglitazone or dimethylfumarate, both used in multiple sclerosis and other neurodegenerative diseases, may be of value 20, 25, 27, 48, 65. Also, the positive effects of curcumin on lifespan and inhibition of oligodendrocyte apoptosis 1, 84 in a jimpy^msd^ mouse [model of PMD with a point mutation 24], and on the locomotor phenotype, astrocytosis, microgliosis and lymphocyte infiltration in the milder overexpressed *Plp1*, PLP‐tg^72/72^ 18, may be related to the anti‐inflammatory, antioxidant properties of the substance.

### Disruption of mitochondrial dynamics in PMD patient's fibroblasts and spinal cords of PLP‐tg^66/66^ mice

Mitochondria are not only highly dynamic organelles that undergo continual fusion and fission events, which are pivotal to maintain their integrity, quality and quantity, but also serve crucial mitochondrial functions and optimize bioenergetic capacity of the cells 38, 46.

In this present work, we identified for the first time the potential role of an impairment of mitochondrial dynamics during the pathogenesis of PMD. We propose that the loss of fission capacity found in human's primary fibroblasts from PMD patients may constitute an underlying pathogenetic event, independent of myelin (or membrane) integrity in this cell type. This supports the notion of a dysregulated mitochondrial dynamics also in spinal cord, based on the increased Fis1 expression and ratios of the main proteins regulating this process. This impaired dynamics may act as a culprit in the mitochondrial dysfunction and ultrastructual morphology abnormalities, such as enlarged mitochondria, observed *in vivo* in the spinal cords of the PLP‐tg^66/66^ mice. Indeed, fission arrest may be an adaptive process to the deficient brain energetic status, preventing mitophagy for preserving residual mitochondrial function, in concordance with findings in patients and mouse models of Alzheimer disease 86. Decreased fission in spinal cords may also account for the observed increased mitochondrial mass and mtDNA, in spite of TFAM downregulation, which may obey to failed compensatory mechanisms in a similar manner as the upregulation of Fis1. Indeed, the seemingly paradoxal increases of mtDNA concomitant to lower levels of TFAM have been described previously 54, thus alternative pathways regulating mtDNA and mitochondrial mass ought to exist 16. Our results suggesting impaired mitochondrial dynamics in spinal cords may impact negatively in myelin formation, a highly energy‐consuming process. Mitochondrial fission occurs at the mitochondria‐associated membranes (MAMs), the structural connection between mitochondria and the endoplasmic reticulum (ER) 35, 36, 62. Another critical role of the MAMs involves initiation of mitophagy process 7, 26. Interestingly, the calnexin (CNX), an ER chaperone which has been implicated in the PMD pathogenesis 30, 72, localize at MAMs and could play a key role in the alteration of fission process 82 and subsequent mitochondrial dysfunction. Indeed, it has been shown recently that silencing of calnexin causes dramatic diminution of mitochondrial fragmentation and reduces the association of the ER with mitochondria and autophagy machinery under stress conditions 82. Finally, this hypothesis is reinforced by the fact that the calnexin deficient mice showed apparent dysmyelination in both CNS and PNS, indicating that calnexin is essential for proper myelin formation 37. Studies focusing on the role of ER chaperones in the malfunctioning mitochondrial dynamics in PMD are thus warranted.

In conclusion, our experiments reveal that *Plp1* overexpression correlates with defective oxidative stress homeostasis and bioenergetic failure: (i) PLP‐tg^66/66^ spinal cords fail to compensate the harmful effects of *Plp1* overexpression, evidenced by oxidative damage to proteins; (ii) PLP‐tg^66/66^ spinal cords present disturbed energetic metabolism, shown by lower ATP levels and abnormal mitochondrial dynamics; (iii) PLP‐tg^66/66^ spinal cords exhibit a defective antioxidant response, evidenced by lower basal levels of catalase and (iv) PMD fibroblasts display mitochondrial dysfunction, demonstrated by higher ROS levels and increased depolarization, lower respiratory control ratio, unbalanced expression of fusion/fission proteins with functionally impaired dynamics and lower ATP levels. These PMD fibroblasts may provide a valuable tool for first screening of drugs targeting mitochondrial function and redox homeostasis.

## Conflict of Interest

None declared.

## Supporting information

Additional supporting information may be found online in the Supporting Information section at the end of the article.

**Figure S1.** Mitochondrial DNA and protein levels are not increased in PLP‐tg66/66 brain at 6 weeks of age. PGC1α, RIP‐140 and DRP1 are induced in brain at the same age. (A) mtDNA content in 2‐ and 6‐week‐old mice and human fibroblasts is expressed as the ratio of mtDNA (cytb) to nuclear DNA (CEBP). (B) CxI subunits NDUFA9 and NDUFB8, CxII, CxIII, CxIV, CxV, VDAC, ACO2 and DLDH protein expression in 6‐week‐old mice. (C) CxI subunits NDUFA9 and NDUFB8, CxII, CxIII, CxIV, CxV, VDAC, ACO2 and DLDH protein expression in human fibroblasts. (D) Relative gene expression of *Pgc*‐1α, *Tfam*, *Nrf1*, *Nrf2* and *Rip*‐140. (D) Relative protein expression of DRP1, Fis1, OPA1 and MFN2. Representative blots are shown. The protein level is expressed as a fold increase of the control and in reference to γ‐tubulin as a loading marker. Two‐week‐old mice, n = 8/genotype; 6‐week‐old mice, WT n = 6, PLP‐tg66/66 n = 7. Values are expressed as the mean ± SD. Statistical analysis was performed using Student's *t*‐test; *P < 0.05, **P < 0.01, ***P < 0.001.

**Figure S2.** Energetic metabolism depletion is not present in brain from 6‐week‐old PLP‐tg^66/66^ mice. ATP levels (A), PK activity (B) and AAT activity (C) are increased in brain in PLP‐tg^66/66^ mice. WT (n = 6) and PLP‐tg^66/66^ mice (n = 7) were used for the study. Values are expressed as the mean ± SD. Statistical analysis was done with Student's t‐test; ***P < 0.001, **P < 0.01, *P < 0.05.

**Figure S3.** Enzymatic antioxidant defense is not altered in brain from 6‐week‐old PLP‐tg^66/66^ mice. Antioxidant enzyme RNA (A) and protein (B) levels were normal, and glutathione reductase activity (C) induced in brain from 6‐week‐old WT and PLP‐tg^66/66^ mice (n = 7/genotype). RNA was quantified with a TaqMan real time PCR system. Relative protein level is expressed as a percentage of control, and in reference to γ‐tubulin as a loading marker. GR activity is expressed as units/mg tissue. Values are expressed as the mean ± SD. Statistical analysis was done with Student's *t*‐test; *P < 0.05, **P < 0.01, ***P < 0.001.
