# Supplementary figures and images for: Oxidative stress and mitochondrial dynamics malfunction are linked in Pelizaeus‐Merzbacher disease
Source: Brain Pathol. 2017 Dec 26;28(5):611–30. doi: 10.1111/bpa.12571 (PMC8028267; doi:10.1111/bpa.12571)

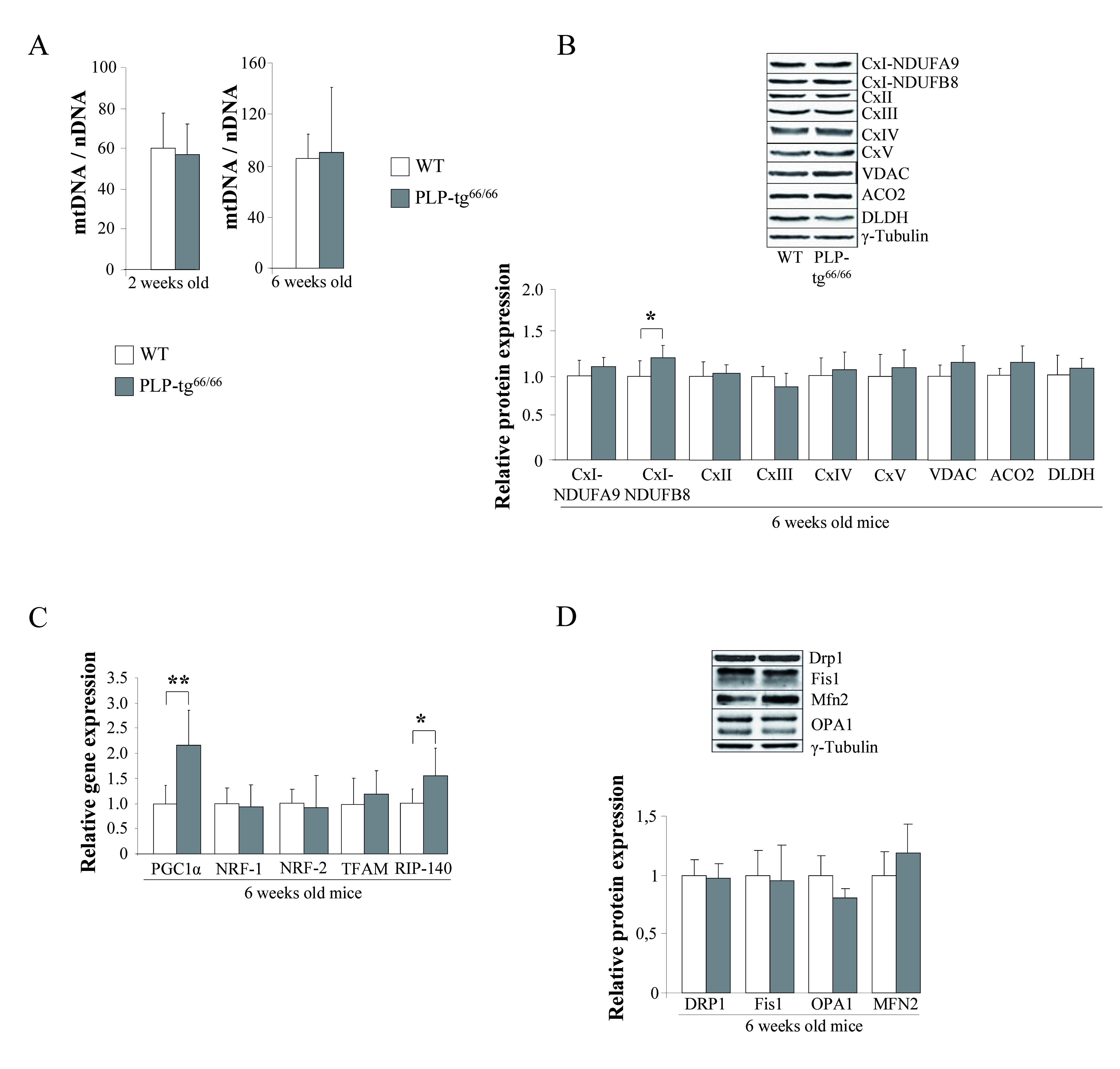

Supplement: Supplementary file 1 — Figure S1. Mitochondrial DNA and protein levels are not increased in PLP‐tg66/66 brain at 6 weeks of age. PGC1α, RIP‐140 and DRP1 are induced in brain at the same age. (A) mtDNA content in 2‐ and 6‐week‐old mice and human fibroblasts is expressed as the ratio of mtDNA (cytb) to nuclear DNA (CEBP). (B) CxI subunits NDUFA9 and NDUFB8, CxII, CxIII, CxIV, CxV, VDAC, ACO2 and DLDH protein expression in 6‐week‐old mice. (C) CxI subunits NDUFA9 and NDUFB8, CxII, CxIII, CxIV, CxV, VDAC, ACO2 and DLDH protein expression in human fibroblasts. (D) Relative gene expression of Pgc‐1α, Tfam, Nrf1, Nrf2 and Rip‐140. (D) Relative protein expression of DRP1, Fis1, OPA1 and MFN2. Representative blots are shown. The protein level is expressed as a fold increase of the control and in reference to γ‐tubulin as a loading marker. Two‐week‐old mice, n = 8/genotype; 6‐week‐old mice, WT n = 6, PLP‐tg66/66 n = 7. Values are expressed as the mean ± SD. Statistical analysis was performed using Student's t‐test; *P < 0.05, **P < 0.01, ***P < 0.001. [file BPA-28-611-s003.tif]

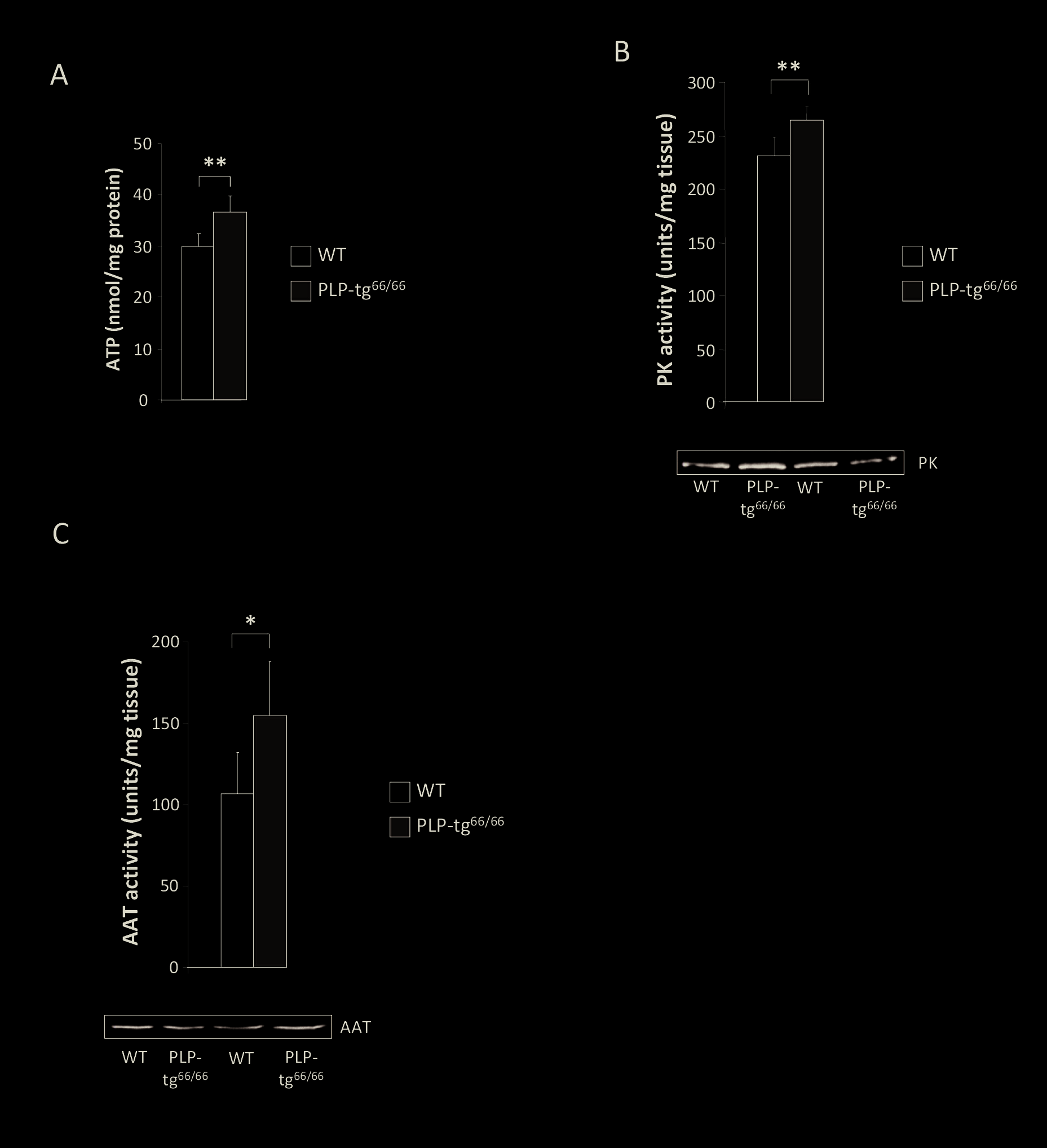

Supplement: Supplementary file 2 — Figure S2. Energetic metabolism depletion is not present in brain from 6‐week‐old PLP‐tg66/66 mice. ATP levels (A), PK activity (B) and AAT activity (C) are increased in brain in PLP‐tg66/66 mice. WT (n = 6) and PLP‐tg66/66 mice (n = 7) were used for the study. Values are expressed as the mean ± SD. Statistical analysis was done with Student's t‐test; ***P < 0.001, **P < 0.01, *P < 0.05. [file BPA-28-611-s002.tif]

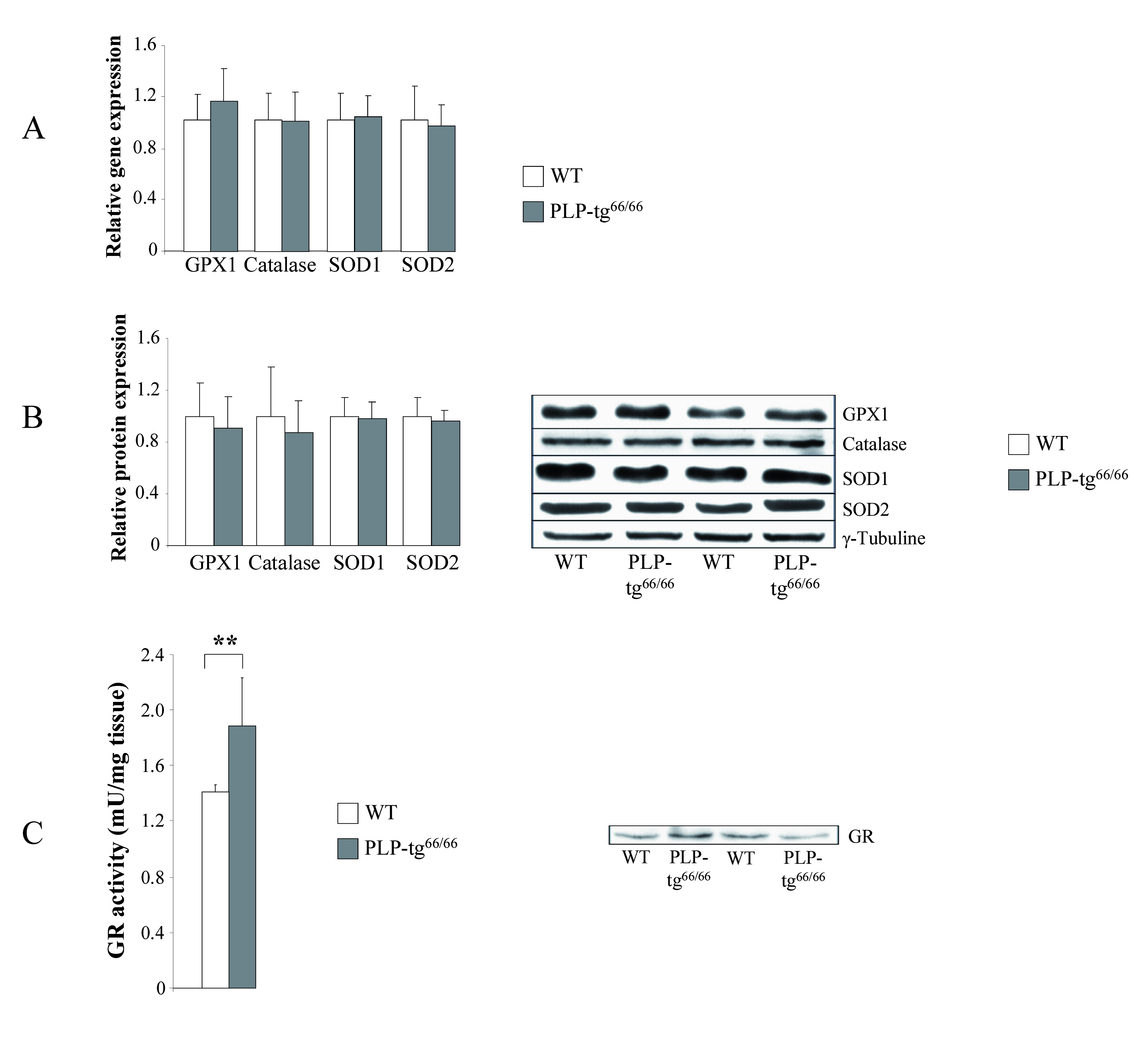

Supplement: Supplementary file 3 — Figure S3. Enzymatic antioxidant defense is not altered in brain from 6‐week‐old PLP‐tg66/66 mice. Antioxidant enzyme RNA (A) and protein (B) levels were normal, and glutathione reductase activity (C) induced in brain from 6‐week‐old WT and PLP‐tg66/66 mice (n = 7/genotype). RNA was quantified with a TaqMan real time PCR system. Relative protein level is expressed as a percentage of control, and in reference to γ‐tubulin as a loading marker. GR activity is expressed as units/mg tissue. Values are expressed as the mean ± SD. Statistical analysis was done with Student's t‐test; *P < 0.05, **P < 0.01, ***P < 0.001. [file BPA-28-611-s001.tif]
